# Caring for AML Patients During the COVID-19 Crisis: An American and Italian Experience

**DOI:** 10.3389/fonc.2020.01689

**Published:** 2020-09-02

**Authors:** Lindsay Wilde, Alessandro Isidori, Gina Keiffer, Neil Palmisiano, Margaret Kasner

**Affiliations:** ^1^Division of Hematologic Malignancies and Hematopoietic Stem Cell Transplantation, Department of Medical Oncology, Sidney Kimmel Cancer Center, Thomas Jefferson University, Philadelphia, PA, United States; ^2^Hematology and Stem Cell Transplant Center, AORMN Hospital, Pesaro, Italy

**Keywords:** acute myeloid leukemia, COVID-19, coronavirus, SARS-CoV2, pandemic

## Abstract

The emergence of severe acute respiratory syndrome coronavirus 2 (SARS-CoV-2) and the subsequent pandemic have impacted every aspect of oncology care worldwide. Healthcare systems have been forced to rapidly change practices in order to maximize the safety of patients and healthcare providers and preserve scare resources. Patients with acute myeloid leukemia are at increased risk of complications from SARS-CoV-2 not only due to immune compromise related to the malignancy but also due to the acuity of the disease and intensity of treatment. These issues have created unique challenges during this difficult time. In this article, we present the approaches taken by two groups of hematologist/oncologists, one in the United States and one in Italy, who have been caring for acute myeloid leukemia (AML) patients in the face of the pandemic.

## Introduction

The coronavirus-19 (COVID-19) pandemic is drastically altering care delivery across all oncologic subspecialties. When safe and possible, delaying treatment is the logical approach in order to decrease viral transmission, preserve scarce resources, and reduce the care burden on an already stressed medical system. However, given the urgent presentation of acute myeloid leukemia (AML) patients, and considering that cure is possible for some, watchful waiting is frequently impossible or inadvisable. In this article, we compare the approach to treating patients with AML at the Sidney Kimmel Cancer Center (SKCC) at Thomas Jefferson University in Philadelphia, PA, United States, and at Marche Nord Hospital (MNH) in Pesaro, Italy, during the COVID-19 pandemic. We place special emphasis on decision-making regarding identifying those patients for whom we can defer treatment until a later, and hopefully safer, date and those for whom we cannot.

## Initial Diagnosis of AML

The diagnosis of acute leukemia in the current era of molecular testing was a challenge even prior to the COVID-19 pandemic. With the added strains of decreased access to in-person and inpatient services, however, it is even more important to get a complete characterization of a patient’s hematologic malignancy prior to starting therapy. It is also of the utmost importance to completely characterize the leukemia and assess the need and potential for future stem cell transplant before starting therapy, as the choice of treatment is being driven both by disease factors and by evidence of and risk of exposure to severe acute respiratory syndrome coronavirus 2 (SARS-CoV-2).

Prior to the current pandemic, two groups presented data at the American Society of Hematology (ASH) 2019 Annual Meeting demonstrating the safety of delaying the onset of therapy until comprehensive molecular and cytogenetic characterization of the disease is obtained. In the first study, the Alliance Leukemia reported on more than 2200 patients presenting for aggressive induction therapy and showed that overall survival did not differ in patients of any age when assessed for days to treatment (0 to greater than 15) (1). That being said, the majority (*n* = 1547) of patients still started treatment between 0 and 5 days, while 447 began between 6 and 10 days, 106 between 11 and 15 days, and only 163 beyond 15 days. Furthermore, the median time from diagnosis to treatment was 3 days in the entire population, confirming that only a minority of patients had a significant treatment delay.

In the second study, the Beat AML Master Trial investigators presented encouraging data in an older, more frail leukemia population, demonstrating that patients who waited for 1 week to enroll in a targeted therapy trial (when one was identified) had superior outcomes to patients who opted to proceed more quickly to standard of care (2). Of note, patients with hyperproliferative disease or DIC were excluded from this study. Also, it is important to remember that AML in the elderly population has different biological characteristics, and a different treatment algorithm, than in the younger population, which limits the applicability of this study (3).

In the setting of COVID-19, and with the support of these data, at SKCC every effort is made to perform a complete diagnostic evaluation in the outpatient setting. This allows patients to remain at home, isolated, for as long as possible prior to starting therapy, thereby decreasing required visits to the outpatient office and hospital and thus reducing both patient and provider risk of exposure to SARS-CoV-2. Routine monitoring of patients undergoing a diagnostic workup requires frequent laboratory evaluations; however, the process has been streamlined, and transfusion practices have been modified, in order to maximize safety. A detailed discussion of our recommended approach to supportive care will be presented in a later section. In addition, telemedicine is utilized for symptom screening, physician visits, and teleconsent for chemotherapy.

The approach at MNH, on the other hand, is to admit patients to the hospital, where rates of SARS-CoV-2 transmission are low, and keep them in isolation until diagnostic results are available. Patient triage prior to admission is performed, when possible, by telephone interview and includes questioning on symptoms (fever, cough, rhinitis, and diarrhea) during the 15 days prior and exposure to COVID-19 positive contacts. Patients with fever are managed in the emergency room, where they perform triage, lab draws, and nasopharyngeal swab. Results of the quantitative polymerase-chain-reaction on nasopharyngeal swabs are available in 6–12 h. When the results are available, patients are managed either outpatient at the hematology department, if negative, or in one of the COVID-19 sub-intensive or intensive care ward of the MNH Hospital.

Regardless of the logistical approach, we agree that interpretation and application of the results of these trials requires caution. Even if the data do support delaying treatment to await the cytogenetic and molecular testing for risk stratification and treatment planning, it is clear that this strategy will not be appropriate for every patient, either during or after the pandemic. For example, anyone with a life-threatening complication such as tumor lysis syndrome, hyperleukocytosis, or neutropenic sepsis should not have treatment delayed. Furthermore, there are some data to suggest that delaying treatment for younger patients may be of detriment. A previous retrospective analysis showed a shorter survival for younger patients (<60 years old) when treatment was delayed by ≥5 days (4). In addition, since the younger population is treated primarily with intensive daunorubicin (or idarubicin) and cytarabine (7+3) induction, and since additional drugs, such as midostaurin or gemtuzumab ozogamicin, are not added until after 7+3 has been initiated, there may not be a benefit to waiting in these patients.

## Testing for COVID-19

Which patients to test for COVID-19 is not straightforward and the guidelines at individual institutions and by national organizations are evolving. Testing should be aimed at protecting patients and the staff. To that end, we recommend universal symptom screening at the front door of the clinic. Additionally, we take the stance that any patient, provider, or staff member could be an asymptomatic carrier and therefore employ universal masking to reduce the risk of spread. In addition, we recommend testing for all patients who are hospitalized or undergoing a medical procedure, and any patient who will be receiving high dose chemotherapy and/or stem cell transplant with the dual goals of delaying therapy, when possible, and protecting staff if therapy is required. Access to testing has been ramped up and is now widely available at SKCC and MNH.

All patients with AML who are not hospitalized, regardless of COVID-19 status, should isolate at home and perform all interventions recommended by the World Health Organization (WHO) to prevent transmission of SARS-CoV-2 outlined in Table 1 (5).

**TABLE 1:** World Health Organization (WHO) coronavirus disease (COVID-19) advice for the public (5).

**Recommendation**	**Specific instruction**
Wash hands frequently	Use alcohol-based hand rub or soap and water for at least 20 s
Maintain social distancing	Maintain at least 3 feet (ideally 6 feet or more) from anyone coughing or sneezing
Avoid touching eyes, nose, and mouth	
Practice respiratory hygiene	Cover mouth and nose with bent elbow or tissue when coughing or sneezing
Seek medical care early for fever, cough, and difficulty breathing	Follow direction of local health authorities
Stay informed and follow advice of healthcare professionals	

## Up-Front Intensive AML Therapy

Treatment of AML in unstable and/or potentially curable patients should not be delayed, despite the increased risk posed by the COVID-19 pandemic. Historically, the care of patients with AML during the induction and peri-induction periods has required prolonged hospitalization due initially to continuous infusion of cytarabine and subsequently for monitoring and management of complications of treatment. With the advent of newer therapies and increased ability to deliver intensive supportive care outside the hospital, this paradigm was changing, even prior to the pandemic. In the present era, as minimizing the risk of SARS-CoV-2 transmission is paramount, it is imperative that we implement care models that minimize patient contact with each other and with healthcare providers.

To this end, the SKCC has adopted the following care processes aimed at (1) providing necessary and appropriate care for patients with AML and (2) managing patients outside the hospital whenever possible. An induction strategy that allows for outpatient treatment is preferred over a strategy that requires hospital admission. We acknowledge that many patients will still require hospital admission for complications of induction therapy.

For patients for whom treatment with 7+3 is required, we opt for early hospital discharge whenever possible. A number of small, retrospective analyses have suggested the safety and reduction in healthcare utilization of early discharge following induction therapy with 7+3 (6–9). In these studies, no early deaths (less than 30 days) were reported. The most frequent adverse event was neutropenic fever, which often required hospital re-admission. In one analysis, however, despite a median of 1.5 hospital re-admissions per patient, the total number of hospital days was still decreased by 30% when compared to inpatient controls (6).

The largest prospective analysis to date on this approach enrolled 178 adults with AML or high-risk MDS receiving induction or re-induction therapy with 7+3 (10). Within 72 h of completion of induction therapy, patients were assessed for eligibility for early discharge based on a number of clinical and logistical factors. One hundred and seven patients met criteria and were discharged within 72 h of completion of induction therapy. Twenty-nine patients were medically but not logistically eligible for early discharge and served as inpatient controls. Despite 93/107 (87%) early discharge patients requiring readmission, primarily for neutropenic fever, they spent a median of 8 days as inpatients (range 0–33) compared to a median of 16 days (range 3–42) for controls. Four early deaths occurred (2.9%), all in the experimental arm, which was not statistically significant (*p* = 0.58) and is comparable to the early death rate for newly diagnosed AML.

For patients where treatment with liposomal cytarabine/daunorubicin is indicated, outpatient induction and consolidation is administered. Since the dosing regimen for liposomal cytarabine/daunorubicin does not require continuous infusion, it is possible to treat patients entirely in the outpatient setting. The SKCC standard prior to COVID-19 was to see patients in the outpatient infusion center days 1–5 where they receive liposomal cytarabine/daunorubicin via standard dosing on days 1, 3, and 5. Laboratory studies and supportive care, such as intravenous hydration and anti-emetics, are administered, as needed, on days 1–5. This practice has been continued during the pandemic. Patients who develop neutropenic fever or other complications of induction are admitted to the hospital, as needed.

The American Society of Hematology (ASH) has suggested continuing to offer consolidative high-dose cytarabine (HiDAC) to patients who achieve complete remission following induction therapy (11). Specifically, it is proposed that the number of HiDAC cycles be reduced to 3 from 4 and that the dose be reduced to 1.5 g/m^2^ from 3 g/m^2^. The SKCC practice is to continue to offer HiDAC consolidation with every effort made to give this treatment at home via in-home infusion; this was also our practice prior to COVID-19. With this treatment, chemotherapy-trained nurses travel to the patient’s home once per day on days 1–3 to infuse the first dose of the day and set up the subsequent infusion each day. Laboratory studies, toxicity assessments, and supportive care are also performed in the patient’s home as indicated.

The approach to the intensive management of AML during the pandemic at MNH has differed significantly from the approach at the SKCC. First, all patients receive induction and consolidation while in the hospital. Accordingly, there is the need to test all patients’ nasopharyngeal swab and a quantitative polymerase-chain-reaction test to detect SARS-CoV-2 infection (RNA) before admission. Ideally, per European Hematology Association (EHA) recommendations, all patients are then re-tested before each treatment cycle, even if they have no symptoms (12). Once hospitalized, all patients must observe a strict reduction of access to visitors to decrease the possibility of becoming positive during the hospital stay.

Symptomatic patients must wait for the results of the swab before hospital admission. If positive, they are hospitalized in a COVID-19 positive environment, with negative pressure when possible. Any decision about treatment is delayed, and the clinical situation is monitored strictly. Hydroxycarbamide is used to manage COVID-19 positive patients as a bridge to intensive therapy when needed.

Young, asymptomatic patients who are SARS-CoV-2 negative receive standard induction mainly with 7+3. Consolidation with cytarabine is also administered in the inpatient setting according to the EHA recommendations; intermediate-dose cytarabine with the 1/2/3 schedule is preferred, as several studies have demonstrated comparable results with HiDAC (13).

Referral for hematopoietic stem cell transplant (HSCT) is a challenging issue in the care of patients with AML under normal circumstances. During the COVID-19 pandemic, issues such as donor availability, testing, selection, and product availability and recipient susceptibility to infection both pre- and post-transplant have become particularly problematic. All transplant registries have been negatively affected by COVID-19, with some experiencing significant difficulty in collecting and exporting products (14). However, delay of transplant, which may allow for emergence of measurable residual disease, is known to negatively impact the survival of patients with AML (9). Therefore, despite the risks and challenges, potentially curable patients with AML at both SKCC and MNH continue to be referred for evaluation for hematopoietic stem cell transplant as indicated. For patients referred for HSCT who become infected with SARS-CoV-2, the European Society for Blood and Marrow Transplantation (EBMT) initially recommended deferral of transplant for at least 3 months; however, this guideline has evolved and the most recent recommendation is to defer transplant for a minimum 14 days (ideally minimum of 21 days) and until the patient becomes asymptomatic and has two negative PCR swabs at least 24 h apart (15).

## Non-Curative AML Therapy

Perhaps the most difficult decision in the treatment of AML during the COVID-19 pandemic is when to initiate therapy in patients for whom cure is either unlikely or impossible with today’s strategies.

In the older (specifically those age >75) and/or unfit populations for whom transplant is unlikely to be considered, careful discussions with patients and their families about the risks of treatment are always paramount; however, these discussions have become even more complicated in the COVID-19 era. At the SKCC, and in the broader United States, for the treatment of AML in the elderly/unfit population, the anti-BCL-2 agent venetoclax in combination with a hypomethylating agent (HMA) has clearly become the standard of care. Initial phase 1 data demonstrated that this therapy is safe and effective at inducing remission, with an overall CR/CRi rate of almost 70%, although this varied significantly by molecular subtype (16). Additionally in this study, the large majority of patients with CRi obtained transfusion independence of red cells (80%) and of platelets (93%) with a median time to response of 1.2 months and best response of 2.1 months. Recently presented abstract data from the phase 3, randomized, double-blinded, multicenter, placebo-controlled study of azacitidine plus venetoclax vs. azacitidine plus placebo confirmed these results and showed an overall survival of 14.7 months vs. 9.6 months, respectively (HR: 0.66, 95% CI: 0.52–0.85, *p* < 0.001) (17). However, in spite of its efficacy, this treatment regimen can have significant side effects, namely profound and/or prolonged neutropenia. This increased risk of infectious complications must be weighed heavily when deciding to initiate treatment in this vulnerable population. Overall, given the strength of this combination to induce remission and, importantly, to provide transfusion—and therefore outpatient infusion clinic—independence, we consider the initiation of HMA and venetoclax in older, unfit patients a priority even during the current pandemic, especially for patients who are transfusion-dependent at diagnosis.

As previously discussed, waiting for molecular sequencing results prior to making a treatment decision is safe and feasible, and oral targeted therapies are particularly attractive treatment options in the wake of COVID-19. The genes for which an oral targeted therapy currently exist include mutated *FLT-3*, *IDH-1*, and *IDH-2* and are present in approximately 50% of AML patients (18). For upfront elderly/unfit patients with a targetable mutation, an oral-only option is a preferred choice at this time, with possible consideration for the potential addition of an HMA in the future as more data emerge (16, 19–21).

Treatment of relapsed/refractory AML during the time of the pandemic also requires difficult discussions with patients and families as to goals of further therapy. If transplant, and therefore potential for cure, is an option, as it would be in an otherwise healthy younger patient, the choice at SKCC is to pursue aggressive re-induction strategies, especially if on clinical trial. However, if there is no potential for transplant, as always, it is preferable to advocate for a lower-intensity, outpatient regimen or palliative care.

The treatment approach for elderly, incurable, and/or relapsed patients with AML at MNH, and throughout Europe, is starkly different and was even before the pandemic. This is mainly because, in the United States, venetoclax + HMAs, ivosidenib, enasidenib, and FLT3 inhibitors are available in clinical practice, but in Europe, they are not. Accordingly, treatment of elderly patients outside of clinical trials and expanded access programs is still very limited. Glasdegib + low dose cytarabine was approved only very recently, and its use in clinical practice is now possible (22). The hypomethylating agents, azacitidine and decitabine, and low dose cytarabine are the only other available treatments. The decision to initiate one of these treatments during the pandemic is largely driven by SARS-CoV-2 status. If positive, it is recommended that treatment be delayed until nasopharyngeal swab RNA-negativity.

As always, for unfit or multiply refractory AML patients, treatment on a clinical trial is preferred and enrollment has continued both at SKCC and MNH during the pandemic. For example, multiple oral-only therapies are under investigation at the SKCC, including dihydroorotate dehydrogenase inhibitors (DHODHi), Axl/Mer pathway inhibitors, among others. These trials, which largely require in-person visits for PK/PD labs only, can be incorporated into a relapsed/refractory patients’ existing transfusion schedule, and therefore provide minimal increase in exposure risk over supportive care alone.

## Transfusion and Growth Factor Support

Providing supportive blood and platelet transfusions is a backbone of leukemia care. However, in the face of a potential blood product shortage and during a time when exposure to healthcare settings must be limited, modifications to institutional transfusion practices have been necessary and implemented both at SKCC and at MNH.

Few data exist regarding optimal red blood cell transfusion thresholds in patients with AML, but data from trials in other patient populations can be extrapolated and applied during this time of need. For example, the TRICC and TRISS trials previously established the safety of a restrictive hemoglobin goal of 7 g/dL in critically ill patients (23, 24). Studies conducted in patients undergoing cardiac surgery and patients with upper gastrointestinal bleeding demonstrated similar safety (24–29).

Perhaps most applicable, however, are the data from the more recently published TRIST trial that randomized 300 patients undergoing either autologous or allogeneic stem cell transplant for a hematologic malignancy to a liberal (9 g/dL) or restrictive (7 g/dL) threshold. The primary outcome of this non-inferiority study was health related quality of life, measured by the validated FACT-BMT score, and the results demonstrated non-inferiority in the restrictive group at all time points (days 7, 14, 28, 60, and 100). There were also no differences in secondary outcomes including bleeding, mortality, infection, or platelet transfusion (30). Taken together, these data support the decision to decrease the red blood cell transfusion threshold from 8 g/dL to 7 g/dL for those AML patients without significant cardiac disease or anemia-related symptoms.

Relatively more data exist regarding platelet transfusion thresholds in acute leukemia. The Platelet Trigger Transfusion Trial was a multi-institutional study that randomized patients with non-APL AML to prophylactic platelet transfusion with a threshold of either 20,000 per cubic milliliter (liberal) or 10,000 per cubic milliliter (restrictive). With respect to the primary outcome of major bleeding episodes, there was no difference between the two arms (31). Other studies have replicated these results (32–34). Thus, through patient and provider reeducation, we have reinforced the safety of a prophylactic platelet transfusion threshold of 10,000 per cubic milliliter and adjusted our care model as needed to support this. For those patients who require frequent platelet transfusions or are transfusion refractory, we also consider the addition of anti-fibrinolytics such as aminocaproic acid or tranexamic acid (11, 35, 36).

Adjustments to protocols for administering growth factors can also be effectively implemented to reduce or eliminate the need for additional in-office visits. G-CSF can easily be given at home via either an on-body injector or daily injection and may reduce the risk of hospitalization due to neutropenic fever or infection.

## Supportive Care

The importance of continuing to provide comprehensive psychological, social, financial, and emotional support for leukemia patients during this time of increased stress cannot be overstated. Transitioning programs that were previously delivered face-to-face to virtual media platforms can be done quickly and with relatively low expense. At the SKCC, multi-disciplinary collaboration between social workers, palliative and supportive medicine providers, financial counselors, and physicians has allowed us to continue to offer psychological, exercise, mindfulness, nutrition, survivorship, and support groups to patients and their family members remotely. Partnerships with local organizations have also served as a means to connect patients to low or no cost WIFI resources, provide technology support, and deliver groceries and other essentials to patients in their homes.

### Real-World Patient Experiences

Fortunately, only one patient with AML, an 82-year-old woman receiving first line treatment with decitabine and venetoclax, has tested positive for COVID-19 at the SKCC. She was treated with supportive care in the hospital and ultimately discharged home; she subsequently resumed outpatient chemotherapy. No patients have delayed induction, consolidation, or transplantation, and there have not been any confirmed COVID-19 related deaths in our AML patient population.

At the MNH, between February 22 and April 26, 21 AML patients were tested for SARS-CoV-2 before admission to the ward. They all tested negative and proceeded with chemotherapy. Furthermore, 3 patients with AML aged 39, 61, and 66 received allogeneic stem cell transplantation (15). Several AML patients with fever went to the ER and were tested for SARS-CoV-2. Two patients aged 51 and 72 with relapsed AML waiting for reinduction chemotherapy tested positive; they were admitted to the intensive care unit and rapidly died due to interstitial pneumonia. When lockdown ended, it became known that 3 other elderly AML patients cared for primarily at MNH had died due to COVID-19 interstitial pneumonia in other hospitals in the Marche region. All AML patients with active disease and SARS-CoV-2 died due to interstitial pneumonia. No AML patient in follow-up at our site had SARS-CoV-2 infection.

## Conclusion

Caring for patients with AML during the COVID-19 pandemic has presented unique challenges. However, through the rapid development of a set of overarching principles to guide care, changes in standard practices have been implemented in order to protect patients and staff. At both the SKCC and MNH, these principles have hinged on (1) continued provision of standard AML treatment for patients who are curable or require immediate intervention; (2) deferment of treatment for stable, incurable patients; and (3) delivery of robust supportive care services through innovative mechanisms. The SKCC approach has also relied heavily on the utilization of outpatient care (including telehealth and home visits) whenever possible, while MNH has opted for an inpatient isolation protocol. We recognize the need to revise and refine these practices as the COVID-19 pandemic evolves, and as we plan for the post-pandemic era of AML care.

## Author Contributions

All authors provided substantial contribution to the conception, drafting, editing, and final approval of this manuscript.

## Conflict of Interest

The authors declare that the research was conducted in the absence of any commercial or financial relationships that could be construed as a potential conflict of interest.
